# The impact of audit and feedback to support change behaviour in healthcare organisations - a cross-sectional qualitative study of primary care centre managers

**DOI:** 10.1186/s12913-021-06645-4

**Published:** 2021-07-06

**Authors:** Anna H. Glenngård, Anders Anell

**Affiliations:** Department of Business Administration, Lund University School of Economics and Management, Box 7080, 2SE-220 07 Lund, Sweden

**Keywords:** Audit and feedback, Change behaviour, Primary care, COM-B, Motivation, Capability, Opportunity

## Abstract

**Background:**

This article addresses the role of audit and feedback (A&F) to support change behaviour and quality improvement work in healthcare organisations. It contributes to the sparse literature on primary care centre (PCC) managers´ views on A&F practices, taking into account the broad scope of primary care. The purpose was to explore if and how different types of A&F support change behaviour by influencing different forms of motivation and learning, and what contextual facilitators and barriers enable or obstruct change behaviour in primary care.

**Methods:**

A qualitative research approach was used. We explored views about the impact of A&F across managers of 27 PCCs, in five Swedish regions, through semi-structured interviews. A purposeful sampling was used to identify both regions and PCC managers, in order to explore multiple perspectives. We used the COM-B framework, which describes how Capability, Opportunity and Motivation interact and generate change behaviour and how different factors might act as facilitators or barriers, when collecting and analysing data.

**Results:**

Existing forms of A&F were perceived as coercive top-down interventions to secure adherence to contractual obligations, financial targets and clinical guidelines. Support to bottom-up approaches and more complex change at team and organisational levels was perceived as limited. We identified five contextual factors that matter for the impact of A&F on change behaviour and quality improvement work: performance of organisations, continuity in staff, size of organisations, flexibility in leadership and management, and flexibility offered by the external environment.

**Conclusions:**

External A&F, perceived as coercive by recipients of feedback, can have an impact on change behaviour through ‘know-what’ and ‘know-why’ types of knowledge and ‘have-to’ commitment but provide limited support to complex change. ‘Want-to’ commitment and bottom-up driven processes are important for more complex change. Similar to previous research, identified facilitators and barriers of change consisted of factors that are difficult to influence by A&F activities. Future research is needed on how to ensure co-development of A&F models that are perceived as legitimate by health care professionals and useful to support more complex change.

**Supplementary Information:**

The online version contains supplementary material available at 10.1186/s12913-021-06645-4.

## Background

Audit and feedback (A&F) in healthcare can be defined as ‘any summary of clinical performance of healthcare over a specified period of time aimed at providing information to health professionals to allow them to assess and adjust their performance’ [1]. Increasingly, performance measurement, including A&F activities, in healthcare has been used to support external accountability, with the overall purpose of improving provider performance and controlling costs [2–4]. This development constitutes a paradigm shift from the perspective of professionals and was inspired by New Public Management (NPM) reforms [5, 6] and trends towards more frequent use of standards in public administration [7]. The use of data and discussions about consequences of clinical audits have partly evolved around the ‘battle’ between non-medical managers and medical professionals around its implications in terms of strengthening public accountability or professional control [3, 8, 9]. In more recent years, policy interest has shifted towards more enabling [10] types of governance and control, allowing for a higher degree of professional autonomy [11, 12].

This article explores the role of external A&F to support change behaviour as perceived by primary care practice (PCC) managers. In particular, we study a) how existing forms of A&F influence different forms of motivation and learning, and b) managers’ views on contextual facilitators and barriers that enable or obstruct change behaviour and quality improvement work. Previous research has identified 17 modifiable design elements of A&F belonging to six categories: the receiver of feedback, type of information, timely delivery, the purpose and rationale, the modality of feedback, and how much information is delivered [13]. A practice of a multimodal form of feedback (combination of written/graphical forms and face-to-face meetings), that givers of feedback are perceived as trusted, comparison with others and use of SMART (Specific, Measurable, Accepted, Realistic and Time based) targets, is recommended by the evidence available [1, 14]. Previous studies have also identified contextual factors that influence effects, not least that the impact of A&F is more visible among providers with poor performance [1, 9, 15]. Of special importance for this study is that the impact of A&F is related to complexity of the targeted behaviour [1]. It is generally more visible when the required change is simple, i.e. when individual doctors and nurses can change independently from others. Examples include adhering to prescription guidelines or treatment guidelines of patients with a chronic disease. Impact is less visible when the required change is more complex, i.e. when change requires collective effort at team and organizational levels.

Despite the rather broad definition of A&F [1], it is predominately seen as an intervention to decrease the gap between evidence and clinical practice [9, 16]. It is assumed that targets reflecting what should be done exist, usually in the form of single interventions and process measures in specialised care [1, 14, 17]. Implicitly, this means that most A&F studies follows a coercive [10] and top-down approach to the implementation and diffusion of clinical guidelines [16, 18]. This approach may improve capability and facilitate the implementation change when the complexity of the targeted behaviour is relatively low. The key problem is seen as aligning performance of healthcare staff with targets. It is important that individual healthcare professionals adhere to stipulated guidelines to enhance equitable and cost-effective healthcare. However, a more comprehensive, systematic and bottom-up driven approach to quality improvement work and innovations is crucial to meet continuously changing healthcare needs, rapid technological developments and scarce resources [19]. This involves a higher degree of complexity in the targeted behaviour at the team or organisational level, typically stimulated by medical progress and professional acceptance [20, 21].

To implement change behaviour in healthcare settings, not only capability, but also opportunity and motivation are critical [22, 23]. Areas of improvement and changes with a low degree of complexity in the targeted behaviour may preferably be generated by individual healthcare professionals in their everyday work. Managerial tasks can be assumed to be of greater importance to foster change with a high degree of complexity of the targeted behaviour. Managers need to coordinate and structure new knowledge, facilitate bottom-up processes and lead the work with routinizing innovations to enhance more complex change [24]. The role of A&F to support managers’ work with more complex change is a less explored research area compared to its role in simple change through the top-down implementation of guidelines [18].

The purpose of this article is to explore if and how different types of A&F support change behaviour in healthcare organisations by influencing different forms of motivation and learning, and what contextual facilitators and barriers enable or obstruct change behaviour in primary care. Our article contributes to the sparse literature on PCC managers´ views on A&F practices. Despite the broad scope of primary care, being ‘that level of a health system that provides entry into the system for all needs’ ([25], p 8) available research about A&F in this setting takes a rather fragmented perspective and predominately concern single interventions and individual doctors’ views, e.g. the role of A&F when implementing guidelines for diabetes care [17]. Our aim is to contribute with knowledge about the impact of A&F to support more complex change. Therefore, we explore PCC managers’ views on all A&F activities that they are involved in. More specifically, we employ a cross-sectional interview study of Swedish PCC managers. We believe that this is a relevant case as Swedish PCC managers are under pressure to continuously implement change to meet the demands from patients, policy makers and payers and, theoretically, the tradition of team-based PCCs combined with fixed payments to providers, facilitates change behaviour [26–28].

## Conceptual framework

Our point of departure is the COM-B framework, which is a health promotion and behaviour change framework and describes how Capability, Opportunity and Motivation interact and generate change behaviour and how different factors might act as facilitators or barriers [23, 29, 30]. Capability refers to individuals’ knowledge and skills that create the capacity to engage in change behaviour. Opportunity refers to the environmental context and resources (physical opportunity) as well as social influences (social opportunity) on change behaviour. Finally, motivation refers to cognitive, emotional, and other psychological processes that lead to change behaviour. The different components may continually influence each other: both opportunity and capability can influence motivation and enacting a behaviour can influence capability, motivation and opportunity. A&F can be seen as a way of influencing these components through the provision of feedback or consequences to health professionals to assess and adjust their performance [1].

### Motivation: commitment to change

Commitment to change can be defined as a force ‘that binds an individual to a course of action deemed necessary for the successful implementation of a change initiative’ ([31] , p 475). Members of an organisation can engage in change behaviour for different reasons. Individuals can commit to change because *they want to* (affective commitment), because *they have to* (continuance commitment) or because *they ought to* (normative commitment). Commitment based on the intrinsic ‘want to’ motives reflects the highest level of commitment to change behaviour. This framework is of particular relevance for healthcare organisations as changes are typically ‘accepted when people are involved in the decisions and activities that affect them, but they resist when change is imposed by others. Policy mandated change is never given the same weight as clinically driven change’ ([22] , p 2).

### Capability: types of knowledge

Capabilities relate to the extent to which resources, including skills, are mobilised to enable the achievement of organisational goals [32]. There are least two types of factors which play a role to facilitate or block innovations in healthcare organisations related to capability, according to previous research [19]. One is the role of evidence, including access to reliable scientific data, not least about the expected impact of changing behaviour in terms of health-related outcome, i.e. ‘know-what’ and ‘know-why’ type of knowledge [33]. The other is the role of partnerships in terms of existing relationships, characterised by trust and mutual support, as a vital starting point of the innovation and a guarantee for its successful implementation, i.e. ‘know-who’ type of knowledge [33]. In more general terms ‘know-why’ refers to knowledge about principles and laws of motion in nature, in the human mind and in society, ‘know-who’ to the kind of knowledge developed and kept within the borders of an organization or team and ‘know-what’ to knowledge about facts and what is usually called information. A fourth type is ‘know-how’, which refers to skills and ability to do something from a theoretical and practical perspective [33].

In regards to implementation of change and innovations, a bottom-up process emphasizes practical experiences, is oriented towards quality improvement work in a local service delivery context and typically produces ‘know-how’ and ‘know-who’ learning [34]. In contrast, a top-down approach, which typically produce “know-why” learning [34], is the predominant perspective on innovation in healthcare. This is consistent with the use of A&F as a coercive intervention to decrease the gap between evidence and practice. It is assumed that targets reflecting what should be done exist, and the purpose is to stimulate change behaviour across recipients of feedback to align actual performance with targets. Innovations, or new evidence, are developed by someone else, frequently by expert committees and health technology assessment (HTA) agencies when it comes to clinical guidelines and by pharma, biotech and medical device industries when it comes to new products [9]. Both bottom-up and top-down approaches generate ‘know-what’ learning [34].

### Opportunity: contextual and social facilitators and barriers to change behaviour

Opportunity refers to physical opportunity afforded by the environmental context and resources and social opportunity ‘afforded by the cultural milieu that dictates the way that we think about things’ ([29] , p 4). Based on previous literature [19, 35], facilitators and barriers in three interrelated levels can be identified: management and leadership, organisational culture and resources, and external environment.

The levels *management and leadership* and *organisational culture and resources* constitute the internal environment of the organisation. A top management with an interest in promoting innovations and coordinating the work with implementation and active promotion and sharing of new ideas and innovations with other organisations function as facilitators to change [36]. This type of management and leadership can also help create an organisational culture characterised by openness towards new ideas and a willingness to carry its potential risks, particularly when the change is not triggered by external factors. A history of previous changes creates organisational receptiveness to change behaviour and the establishment of organisational norms and beliefs guiding the generation of new ideas [19]. With regard to organisational resources, a good staffing situation, adequate financial resources and time are fundamental facilitators to change behaviour. An organisational barrier to change in this area is heterogeneity between staff and management, e.g. if decision-makers and potential innovators belong to different professional groups, change could be blocked because staff have to convince managers of the usefulness of implementing it [19]. With regard to the *external environment*, detailed assignments and legislation impede flexibility and constitutes barriers to change behaviour as well as reoccurring interventions imposed by external actors as this might create a general fatigue towards new initiatives [35].

To sum up, A&F can be seen as a way of influencing motivation, capability and opportunity for change behaviour in healthcare through the provision of feedback or consequences to health professionals to assess and adjust their performance. Contextual opportunity barriers, e.g. a lack of resources to enact a behaviour, are difficult to influence by A&F activities, However, such contextual opportunity barriers might influence the impact of A&F activities. For example, recipients who lack resources necessary to enact a behaviour, suggested by givers of feedback, are less likely to find performance feedback relevant, compared to recipients with adequate resources [23]. Nevertheless, for A&F to have an impact on change behaviour, it is important for givers of feedback to be aware of and consider factors acting as facilitators and barriers to change. Otherwise, it will be difficult to tailor feedback messages in a way that it is meaningful and usable for recipients [15, 23, 30].

## Materials and methods

### Context

The Swedish healthcare system is highly decentralised. Primary care is organised in 21 regional welfare markets with choice of PCC for individuals and competition among private and public providers. There are about 1200 PCCs in Sweden, whereof about 40% are private, predominately for profit. The regions control the establishment of PCCs by regulating the local requirements (financial, organisational and quality requirements) that PCCs have to comply with in order to practice primary care with public funding. By law, the same requirements apply to all public and private providers [37]. Healthcare providers also have to adhere to national guidelines and recommendations, issued and monitored by government agencies and national committees and expert groups (e.g. the Swedish strategic programme against antibiotic resistance, STRAMA). In each region there is also a local pharmaceutical committee, providing guidance and support for appropriate use of pharmaceuticals in general. PCCs typically consists of 40–50 staff with a mix of professional competencies, e.g. general practitioners (GPs), nurses with different specialisations and physiotherapists. Theoretically, the tradition of a team-based PCC structure facilitates change through opportunities for flexible use of resources, e.g. task shifting between GPs and nurses [28, 38]. Payment to PCCs consists predominately of fixed capitation, combined with a comprehensive financial responsibility for the need of primary care among registered patients [26]. Financial incentives too create opportunities for change as they do not prevent a flexible use of resources [27]. Each PCC must have an appointed managing director (referred to as PCC manager in this article), in charge of operations. PCC managers come from different professional backgrounds, e.g. GP, nursing or administrative [39]. In case the PCC manager has another background than GP, there must also be an appointed clinical director, a GP, with the overall medical responsibility for the activities at the PCC. Contracts are signed between the PCC manager and a contract manager in each region.

### Study design and data collection

The source of data was semi-structured interviews with PCC managers in five regions. We used a cross-sectional design and a purposeful sampling to identify both regions and PCC managers, in order to explore multiple perspectives. The selection of the five regions was done primarily with respect to variation in population size, number of PCCs and mix of public and private PCCs, as these factors may lead to variation in the types of A&F activities used. In total, we interviewed 35 individuals. First, we interviewed key informants in the regions to get a description of existing A&F activities; 12 respondents in total, all with long experience as contract managers or medical advisors. Second, we interviewed 23 PCC managers of 27 PCCs (some managers were responsible for more than one PCC) about their views on A&F (Table 1). The selection of PCCs was done with respect to variation in ownership (public, private single, private group), size (PCC list size) and background of PCC manager (GP/nurse/other).

**Table 1:** Interviewed PCC managers and PCC characteristics

Interviewee	PCC(s) represented^a^	Background of manager	Size of PCC^b^
Region A (Large^c^, 50% private PCCs)			
1	1 Private, group	Nurse	Medium
2	2 Public	Nurse	2 small
3	2 Private, small group	GP	1 large/ 1 small
Region B (Large, 50% private PCCs)			
1	1 Private, group	Other	Large
2	1 Private, group	GP	Large
3	1 Public	Other	Large
4	1 Public	Nurse	Large
5	2 Public	Nurse	1 large/ 1 medium
6	1 Public	Nurse	Large
7	1 Public	GP	Large
8	1 Private, single	Other	Large
Region C (Small^c^, 20% private PCCs)			
1	1 Public	Nurse	Large
2	1 Public	Nurse	Medium
3	1 Public	GP	Large
4	1 Private, group	Nurse	Large
Region D (Small (40% private PCCs)			
1	2 Public	Nurse	1 large/ 1 small
2	1 Public	Nurse	Large
3	1 Private, small group (nfp)	Nurse	Small
Region E (Small, 50% private PCCs)			
1	1 Public	Nurse	Medium
2	1 Public	Nurse	Medium
3	1 Private, group	GP	Medium
4	1 Private, small group	Nurse	Large
5	1 Private, group	Nurse	Large
23 PCC managers	27 PCCs		

^a^single = one PCC only; small group = 2–5 PCCs, group = 6+ PCCs. ^b^PCC list size: Small < 5000; Medium 5000–10,000; Large > 10,000; ^c^Large = > 1000′ inhabitants & > 150 PCCs; Small = < 500′ inhabitants & < 50 PCCs

Both researchers participated in the interviews with key informants in the regions and PCC managers (24 and 11 interviews respectively). We use pseudonyms for the regions (letters) and managers (numbers) to anonymise respondents. The interviewees were invited to participate via e-mail. Two remainders were sent to those who did not respond within 14 and 28 days respectively. All interviewees were informed that participation was voluntary and they could discontinue the interview at any time. The interviews were open in nature, giving the respondents room to reflect upon the subject investigated and engaging in discussions with the researchers. We used an interview guide, based on our theoretical framework, to ensure that relevant aspects were covered. Each interview lasted between 50 and 80 min. All interviews were conducted between April and November 2019 at the interviewees work place or over phone. All interviews were recorded and transcribed verbatim.

### Analysis

Directed content analysis was used to guide the analysis of the collected data [40]. First, we reviewed the transcripts from the interviews with key informants in the regions to provide a description of different A&F activities based on important dimensions identified in previous research [13, 14, 41] (Table 2).

**Table 2:** Types and design of A&F activities in the studied regions: summary from interviews with key informants

Giver & recipient of feedback	Purpose of A&F	Type of data	Modality of feedback
Region as payer and regulator.			
Giver: contract manager and often medical advisor (GP). Recipient: PCC manager and key staff.	Control of compliance to contractual obligations. In combination with some support to learning and innovation at both practice and regional levels through dialogue and information exchange.	Combination of measures at practice level. Structural requirements regarding facilities, staff, compliance towards opening hours, collaboration agreements, patient satisfaction, adherence to clinical guidelines, waiting times etc. Targets linked to contractual obligations. Some targets related to quality measures.	Modality varies. Multimodal with face-to-face meetings in several regions (sometimes using Skype), usually annual or bi-annual. Data rarely available in-between feedback meetings.
Regional pharmaceutical committees & the national The Swedish strategic programme against antibiotic resistance (STRAMA*).			
Giver: usually GP or senior professional. Recipient: PCC manager and individual prescribers (GPs).	Control of compliance to regional recommendations and evidence-based national targets. In combination with some support to learning and innovation through educational seminars, dialogue and exchange of information.	Process measures related to prescriptions and use of medicines at practice level and individual prescriber level. Targets linked to clinical guidelines, recommended drugs and restrictive use of antibiotics.	Usually multimodal. Face- to-face meetings (sometimes using Skype), usually more frequent than on an annual basis. Data available in-between feedback meetings.
Owners of PCCs (public or private)			
Giver: controller and/or medical advisor (GP) Recipient: PCC manager.	Control of compliance to contractual obligations in combination with focus on efficiency measures.	Combination of measures at practice level and to some extent at individual staff level. Focus on costs and volumes of care in addition to measures used by the region. Targets focusing on costs and output volumes, waiting times and quality measures.	Frequency varies, often monthly. Face- to face meetings. Data sometimes available in-between feedback meetings.

Second, we analysed the transcripts from the interviews with PCC managers. As a starting point, the transcripts were reviewed to get an overall picture of their views on different A&F activities to support change behaviour in their organisation. Thereafter we analysed the transcript thematically, focusing on categorizing and describing the findings with regard to our conceptual framework:

- The impact of different types of A&F on motivation, operationalised as different types of commitment to change (want-to, ought-to, have to).
- The impact of different types of A&F on capabilities, operationalised as different types of knowledge generation (know-what, know-why, know-how, know-who).
- The occurrence of contextual facilitators and barriers for opportunity to change behaviour, and its relation to different types of A&F.

Transcripts were also analysed with regard to possible differences in perceptions among managers. The quotes presented have been chosen to illustrate how managers with different backgrounds and representing PCCs with different characteristics perceive the impact of A&F and the opportunity to change behaviour in their organisations.

Both researchers reviewed the transcripts independently and then we discussed findings and reached consensus about the empirical results. Our views did not differ significantly but rather served to complement and nuance findings and identify suitable quotes.

## Results

### Description of A&F activities

Although slightly different A&F activities are used in the five regions, three main forms could be identified: from owners (public and private), payers/purchasers (regions) and regional and national committees focusing on appropriate use of antibiotics and other pharmaceuticals. The three types are linked to differences in purpose and type of data but also in modality (see Table 2).

Some variation in for example the modality of A&F of could be noted across the five regions (frequency of feedback messages, use of face-to-face meetings). However, differences were minor and, in line with our stated purpose, our focus is on PCC managers perception at the aggregate level.

### Perceptions about different types of A&F to support change behaviour

According to the PCC managers, A&F from the region as payer and regulator is targeted at compliance to contractual obligations. The giver of feedback is usually a contract manager at the region, sometimes accompanied by a medical advisor, and the recipient is the PCC manager and sometimes the clinical director and additional key employees. Data on performance is presented on an aggregated level for the PCC, in comparison with targets and/or the mean of a group of providers in the region. Feedback is given once a year or with even longer time intervals. Most of the interviewed PCC managers in all regions expressed a view that regions have an interest in supporting providers to meet requirements in contracts and facilitate for them to deliver care in accordance with regulations, guidelines and targets. Sanctions were perceived as a last resort if PCCs fail to meet targets. This type of A&F provides PCC managers with information about deviations from targets and sometimes about good examples regarding how other PCCs have solved similar challenges. However, A&F from the region was not perceived as useful to support quality improvements in a broader sense because data and feedback is not closely related to daily operations:

*‘The annual feedback is too general. An inspection! Not close to operations. It has never crossed my mind that it should be to improve quality. Maybe they SHOULD be inspectors. And then support clinical improvement in another way. [ … ] It is not a deeper support in itself. But the intention is good!’ (Region C #4)*

A&F from the owner is targeted at reaching contractual obligations, financial targets and – to various degrees – clinical targets, according to the interviewed managers. Both public and private owners give feedback on performance on an aggregated level for the PCC, in relation to the mean of the PCCs belonging to the same owner or more transparently with other providers when the data is available. Data on e.g. volumes of services produced is often also available at the level of each employee. The giver of feedback is usually a controller, often accompanied by a medical advisor, and the recipient is the PCC manager and sometimes the clinical director and additional key employees. Feedback is given more frequently from owners than from the region, i.e. quarterly or monthly. According to the managers, the owners have an interest in supporting providers to meet requirements in contracts and facilitate for them to deliver care in accordance with guidelines that meets the demands of their patients but also focus on financial efficiency measures. Similar to A&F from the region, the focus is to help them reach targets. Generally, this type of A&F is targeted primarily at financial aspects and to a lesser extent at quality aspects, according to the interviewed managers. A&F from owners was perceived as having more impact on change behaviour than A&F from the region as data is more closely related to operations and feedback is given more regularly. The impact was perceived as more relevant for productivity (volumes) and financial aspects than quality aspects, although the latter was also mentioned.

*‘Follow-up of the number of lab samples and X-ray examinations. Much of that is the responsibility of our clinical director to monitor throughout the year. The data is transparent so that you can see exactly at the individual level who has ordered which samples or examinations. (I: Is it mainly about using resources in the best way?) Yes, exactly.’ (Region C #2)*

*‘We have a balanced scorecard that we monitor. [ … ] We have data for all PCCs and it is presented once a month. All public PCCs in our area. So, this is a very good document to find out how others are doing and to be able to use good examples. [ … ] What does our telephone access look like? It has been the most prioritized question, that if you call your PCC, you should get a prompt reply. How many home visits do we make? How many coordinated individual plans, have we made? How are we doing in journal writing? What does the sick leave look like? Short absence, long absence. And what about the financials?’ (Region B #3)*

A&F from regional pharmaceutical committees and the Swedish strategic programme against antibiotic resistance (STRAMA) is targeted at clinical data and medical evidence. Data is presented at both aggregated level for the PCC and at the individual prescriber level. Feedback is given rather regularly, often quarterly, and data is normally available to PCCs in-between feedback meetings. The givers of feedback are senior professionals with a clinical background and the recipients of feedback are GPs. Adherence to pharmaceutical guidelines and a restrictive use of antibiotics is something that providers both have to and want to comply with because it has direct benefits for individual patients and society (avoiding antibiotic resistance). This type of A&F was perceived as useful to support the quality and adherence to guidelines and appropriate use of antibiotics, according to the managers. Two interconnected factors of importance to support change behaviour were identified: Firstly, the data used by STRAMA and regional pharmaceutical committees was perceived as being close to daily operation, and feedback messages was considered updated, concrete and tangible. Secondly, the legitimacy of the giver and the relevance of the feedback messages was perceived as high, since givers (mainly senior GPs) had similar professional backgrounds as recipients.

*‘That A&F from STRAMA is more useful [than A&F from the region], I think, it is because it concerns things you actually do every day. It's a little more diffuse things that the others give feedback on. It's not just about what I do. It is more diffuse because it becomes easier to hide in the crowd [when it is reported on an aggregated level].’ (Region C #1)*

*‘I can follow these [prescription data] continuously month by month and show the doctors. So, I benefit greatly from this pharmaceutical committee, not only the feedback meetings but also the statistics that they provide. We can get it at the prescriber level so it is very valuable. [ … ] Somehow, it is perceived as evidence what the pharmaceutical committee says. It has a good reputation among doctors.’ (Region E #4)*

### Perceptions about data used in A&F

The results from the interviews suggest that the professional background of PCC managers matters for the type of data demanded and used. The overall objective of the PCC was described in similar words by all managers irrespective of background: to provide good quality primary care corresponding to the needs and expectations of outpatient care in the population. But the use of data to reach that objective seems to differ. Managers with a GP background and who also work in clinical practice are generally more inclined to use and act upon outcome in performance measures on clinical quality in the daily operations, according to the interviews:

*‘It's like this that I, as a manager, am deeply involved in the operations, you can discuss the good and the bad with that. My identity is mainly a doctor and that means that I am close to the daily work all the time and really it is important to do a good job so that the patients are satisfied.’ (Region E #3)*

*‘Our focus has been on improving the clinical experience for patients. Then there is often a side effect of patients being satisfied. It will be a better working environment and this in turn will improve the financial result. We have never had profit as a main target.’ (Region A #3)*

PCC managers with a non-GP background who did not split their time between administrative and clinical work focused more on other types of measures in their daily work and then involve the clinical directors in clinical improvement work.

*‘We do this [monitor the number of visits per doctor] every month. And we have included a number of visits in the budget as an income. Then we have formulated it as for example 1250 visits per month per full time doctor. Based on that, you have a budget depending on the number of doctors, the number of nurses you have, then you follow it up every month.’ (Region E #2)*

### Limited support from a&F to bottom-up driven change

All interviewed PCC managers explained that the most important work with bottom-up driven change is generated at the PCC, stemming from identified needs related to the operational work and where the manager plays an important role in encouraging staff and coordinating the work.

*‘For improvement work, it is access to data and the small group with close collegial feedback that matters. Internal leadership and management are more important than someone coming from outside for the continuous improvement work. Internal driving forces are the most efficient.’ (Region C #3)*

*‘It is always best if [the improvement suggestions] comes from the employees themselves. But sometimes you are forced to do something as a manager. We have structured a system for improvement plans. Like a paper with what you want to change, the purpose, who is responsible, what date and when it should be followed up. So, we put it in a folder and go through it every now and then at staff meetings. This way we can also follow up when we have a planning day what changes have been made. Because there has been a feeling that we are talking about it, but nothing is happening.’ (Region B #7)*

*‘I am always open and invite staff to dare to come up with ideas and try them. Even though I do not always believe in the idea, I let them test. … Sometimes it is a failure … and sometimes it is actually a success and then that new idea becomes routine, so to speak.’ (Region B #3)*

### Facilitators and barriers for opportunity to change behaviour

A good overall performance of the PCC was described as a crucial factor for the ability to work with continuous quality improvements and innovations in a proactive manner. A good performance, which is commonly associated with a good continuity in staff, creates slack in the organization. When human and financial resources do not have to be dedicated to recruitment of staff and ensuring that daily operations run according to guidelines in the short run, there is more room for using performance data in a proactive manner in the organisation. Hence, when providers are performing well, A&F is of less impact to force commitment to change among managers. Then, the feedback becomes more of a confirmation that everything works well at the PCC.

When PCCs are performing poorly there is more need for change but respondents also pointed towards less room for strategic thinking and proactive work in practice:

*‘When there is such a negative spiral in a PCC that everything goes awry, for some reason all routines disappear. That's very strange, I think. But this was the case anyway, so everything has had to be rebuilt. [ … ] I have worked a lot with that [setting up routines].’ (Region B #5)*

When providers face problems reaching financial and other targets and adhering to rules stipulated in contracts, A&F has a greater impact for PCCs. Then, the feedback is perceived as something that managers cannot choose to ignore but have to comply with to achieve minimum requirements to avoid sanctions. The approach to change becomes reactive, as a response to problems identified by a pharmaceutical committee, the owner or a contract manager at the region. Providers then have to implement change to reach requirements stipulated in contracts and agreements and/or guidelines. Discussions with givers of feedback are important to pinpoint deviations from targets and to help identify solutions to problems on how to reach minimum requirements, often through adopting solutions developed by others.

*‘If you reach targets, then everything is okay. If you perform below targets, you will get a yellow if you imagine the traffic light. If it is down the wrong pipe completely, it will be red. Then you risk a deduction in pay and you have to submit an action plan on how to improve performance.’ (Region D #2)*

The size of PCCs is an important contextual factor for the impact of A&F and opportunity to implement change. Managers of smaller PCCs expressed a greater demand for an external actor to compile data for them and present in an easily understood format. Simply having registers where PCC managers and other staff can retrieve the data themselves is not enough, according to several interviewees for smaller PCCs. Larger PCCs have better opportunity to devote time and resources to derive, compile and utilise data themselves and to make meaningful comparisons across patient groups and/or individual prescribers, according to the interviewed managers.

*‘Easier to work with changes and improvements if you are a larger PCC. Of course, it is easier to devote [staff] if you are about six ordinary doctors compared to one or two.’ (Region C #3)*

The interviewed managers also reflected upon facilitators and barriers for opportunity to change behaviour related to type of owner. The general view expressed was that private ownership is associated with more flexibility and quicker decision making, which facilitates the implementation of change.

*‘It is light years better to work privately, I must say, which I never thought. It is much faster to make changes and shorter paths to decision. If we see that something does not work here than we do not continue to work like that. Then we change it and we can do it very quickly.’ (Region A #1)*

*‘In a way, there are challenges [to be a public PCC]. There are a lot of things that I just have to do. I have to follow a lot of rules and policies. I cannot choose premises and there are a lot of other things I cannot choose. Then on the other hand in a large private group you cannot choose that much either. But of course, it would have been more flexible to run a single private practice.’ (Region B #7)*

## Discussion

The interviewed PCC managers perceived the three forms of A&F identified as coercive top-down approaches to the implementation of contractual obligations and clinical guidelines. Our results suggest that that this type of external A&F provide limited support to complex change but can have an impact on change behaviour with a low level of complexity in the targeted behaviour. A&F from owners and purchasers, which dominated, can have an impact through ‘know-what’ and sometimes ‘know-why’ types of knowledge [33, 34] and commitment primarily based on ‘have-to’ motives [31]. A&F from regional pharmaceutical committees and STRAMA, focusing evidence based clinical guidelines, can have an impact also on ‘want-to’ commitment and ‘know-how’ types of knowledge. Important factors for A&F to have an impact on the latter type of motivation and capability is that data is perceived as updated, reliable and close to daily operations and that the giver and recipient of feedback have similar professional backgrounds. Hence, while previous research finds that access to reliable scientific data and relationships characterised by trust and mutual support between actors within an organisation play a role to facilitate change [19], we find that these factors function as facilitators to change also between givers and recipients of external feedback.

Access to reliable and updated data is fundamental to support managers with any type of change, according to views from managers. Different types of data support different types of change, weather it is related to clinical quality at treatment level or change at team or organizational levels. We find indications that the professional background of managers matters for the type of data used. Managers with a professional background as a GP and who share their time between administrative and clinical work seem to rely more on measures of clinical quality and patients’ experiences, and to look at production data and volumes only if needed. If the clinical experience is good, patients will be satisfied, which will lead to a better working environment, more satisfied employees and in the end also a good financial result. Managers with a non-GP background and who do not work clinically in parallel with managerial duties seem to start in the other end; with measures and volume-targets that can and has to be produced given the resources available, in order to meet the expected needs and demands of purchasers of care and patients.

Our results show that several contextual factors matter for the impact of A&F and enable or obstruct change behaviour. Poor performance may function both as a barrier and a facilitator to change behaviour. On the one hand, similar to previous research [1, 15], we find that the impact of A&F on change behaviour is greater when providers are performing poor. When providers do not reach minimum requirements stipulated in contracts and agreements and/or guidelines, they are forced to implement change in response to identified deviations in order to avoid sanctions. Thus, poor performance is associated with strong ‘have-to’ motivation to change behaviour. On the other hand, the opportunity for change involving a higher level of complexity in the targeted behaviour increase with good performance and a good continuity in staff – two factors that are commonly interrelated. Such conditions create slack in the organisation and more room for implementing change in a proactive manner based on ‘want-to’ motives rather than in a reactive manner based on ‘have-to’ motives, as less resources have to be dedicated to manage daily operations and ensuring adherence to minimum requirements.

Other contextual factors related to opportunity to change behavior is size of the organisation and staffing situation. According to our results, the opportunity to make meaningful use of data and to dedicate resources to derive and interpret data is contingent upon size and a stable staffing situation. Managers of larger PCCs with a stable staff situation have better opportunity to devote resources to derive, compile and utilise data themselves compared to managers of smaller PCCs, who expressed a greater need for external support to present data in an easily understood format. It is more difficult to compare performance across patient groups or individual prescribers within a PCC if the volume of patients and services is low. Hence, the impact of A&F on ‘know-what’ and ‘know-why’ types of knowledge was found to be greater in smaller organisations. Previous research has also shown that size is related to the likelihood of adopting management innovations in public sector organisations in general [42–44]: The need for formal controls increases with size, and the capacity to use different controls increases with size as the administrative capacity to adopt and adjust controls is related to the number of staff.

Ownership was also mentioned as a contextual facilitator or barrier to change behaviour. The general view expressed by managers of both public and private PCCs was that private ownership is associated with more flexible leadership and management, e.g. quicker decision-making routines, as well as a more flexible external environment, e.g. less detailed requirements, which enables change behaviour. Hence, it is not ownership per se but the degree of organisational receptiveness to change behaviour [19] that constitutes a barrier or facilitator to change behaviour, according to our results.

Previous research concludes that a fundamental requirement for a successful implementation of change in healthcare organisations is that medical professionals are committed to change behaviour [20, 22]. Similar to previous research, our empirical study of PCC managers’ views suggests that the impact of external A&F to support bottom-up driven and more complex change is limited [15, 19, 30]: Change motivated by ‘want-to’ commitment and knowledge characterised by ‘know-who’ and ‘know-how’ is the result of implementation of ideas identified by individual healthcare professionals in their everyday work. The interviewed managers expressed that they try to foster such work through encouraging the generation of new ideas within the organisation, coordinate and structure new knowledge, and allocate resources to the work with routinizing innovations, i.e. afford physical and social opportunity to change behaviour [19, 29, 35].

### Strengths and limitations

A strength with this study is that we explore multiple perspectives of the impact of A&F and thereby identify different forms of motivation and learning as well as facilitators and barriers to change behaviour in health care. Moreover we find that primary care managers share similar views on the impact of A&F although characteristics of the PCCs differed, which suggests that our results can be generalised at least in the Swedish setting. Some variation in different A&F activities in the five regions were observed, for example in how often feedback was given, if data was available in-between feedback meetings and if the feedback was given face-to-face or using Skype or a similar media was noted. However it was not possible to further explore the impact of this variation due to limited sample size. Finally, although the use of open-ended questions gave the respondents room to reflect upon the subject investigated, there is always a risk of researchers affecting responses and the reproducibility of results with the chosen methodological approach.

## Conclusions

Our results suggest that external A&F, perceived as coercive by recipients of feedback, can have an impact on change behaviour through ‘know-what’ and ‘know-why’ types of knowledge and ‘have-to’ commitment but provide limited support to complex change. Similar to previous research [23], the identified contextual opportunity facilitators and barriers – size, performance, staffing situation and internal and external flexibility – consist of factors that are difficult to influence by A&F activities. As these facilitators and barriers vary across providers, for A&F to have an impact on complex change, feedback messages need to be carefully tailored to fit recipients’ opportunity to engage in change behaviour [15, 23, 30]. Moreover, the recipients of feedback need to have an active role in A&F activities to avoid gaps between the givers’ information and intentions and recipients’ motivation, capability and opportunity to engage in change behaviour [30]. A&F should enable providers themselves to define the most relevant problems, changes needed and quality improvement initiatives. This approach to A&F is in sharp contrast to a coercive approach, where targets are formulated top-down and fulfilment of those targets determine the distribution of rewards and sanctions across providers [45]. Future research is needed on how to ensure co-development of A&F models that are perceived as legitimate by health care professionals and useful to support more complex change.

## Supplementary Information

**Additional file 1.** Interview guide.

## Data Availability

The dataset generated and analysed in the study consists of transcripts from interviews with PCC managers and key informants in five Swedish regions. The transcripts are not publicly available but are available from the corresponding author on reasonable request and permission from the interviewee(s).
