# Supplementary material for: The impact of audit and feedback to support change behaviour in healthcare organisations - a cross-sectional qualitative study of primary care centre managers
Source: BMC Health Serv Res. 2021 Jul 6;21:663. doi: 10.1186/s12913-021-06645-4 (PMC8258937; doi:10.1186/s12913-021-06645-4)
Supplement: Supplementary file 1 — Additional file 1. Interview guide. [file 12913_2021_6645_MOESM1_ESM.docx]

#### Interview guide - PCC managers

####

#### Background information about manager

- Professional background (nurse, doctor, managerial, etc.)
- Number of years at current position
- Number of years in region
- Experience from other sectors/settings

*Background information about PCC*

- Public/private
- Size – patient and staff
- Patient characteristics
- Staff mix, and staff situation
- Overall performance of PCC and how it has developed over time.

#### Which actors are involved in A&F activities/from which actors do you get feedback?

#### What is your opinion about the purpose or intention of A&F activities from different actors?

- Enabling (e.g., enhance motivation)/ coercive (e.g., assure adherence to guidelines)

#### Describe the A&F activities from each actor.

- Modality/frequency/time lag/ type of data?
- Who gives feedback?
- Who receives feedback?

#### How useful is A&F from different actors?

- For you as manager?
- For daily operations?
- To facilitate simple and complex change?
- What factors make A&F more or less useful to support change?

#### What is the role of A&F to reach different objectives and targets?

- Are there specific quality targets and other targets?
- Who set targets and measures (external/internal)?
- How are targets set (bottom up/top down)?

#### What is the role of A&F in improvement work in general?

- How do you work with innovations and quality improvements?
- What factors facilitates or obstructs quality improvement work?
- What data do you use to follow up on quality?
- What factors explain differences in quality improvement work between PCCs?
- Who is responsible for quality improvement work at your PCC?
- What is the role of quantitative versus qualitative data?

#### Could you describe an ideal A&F model?
